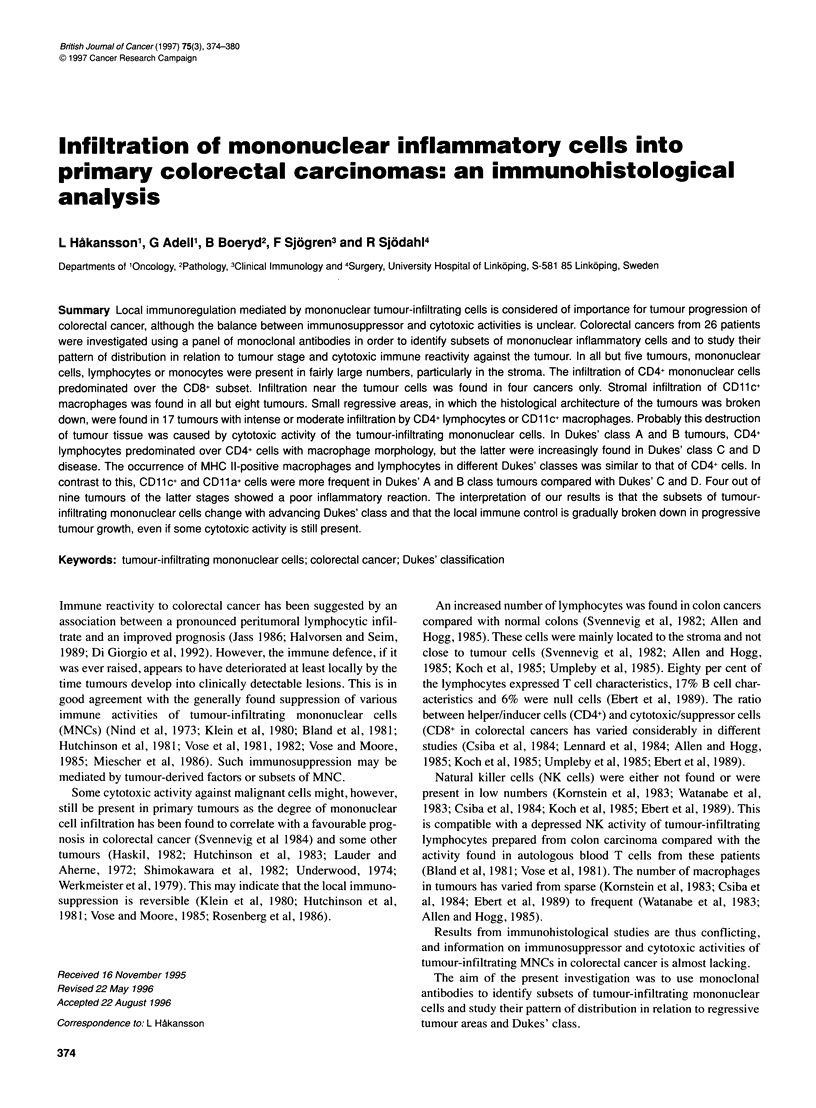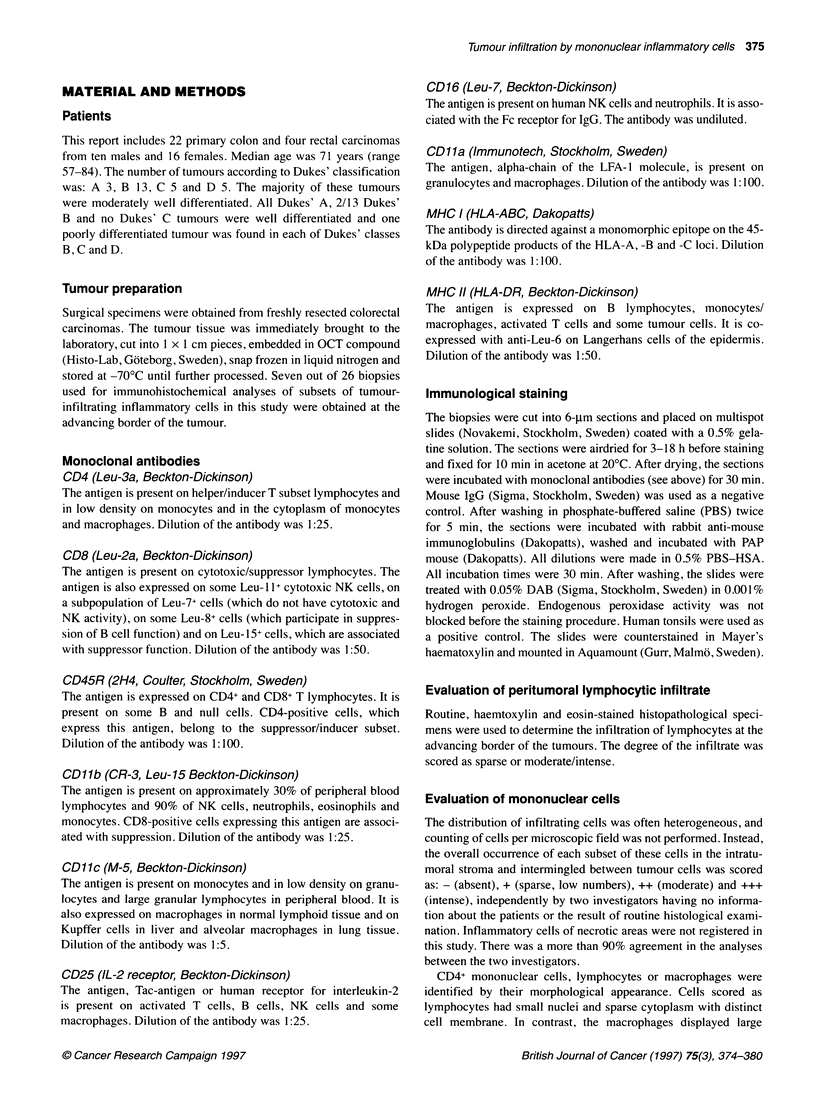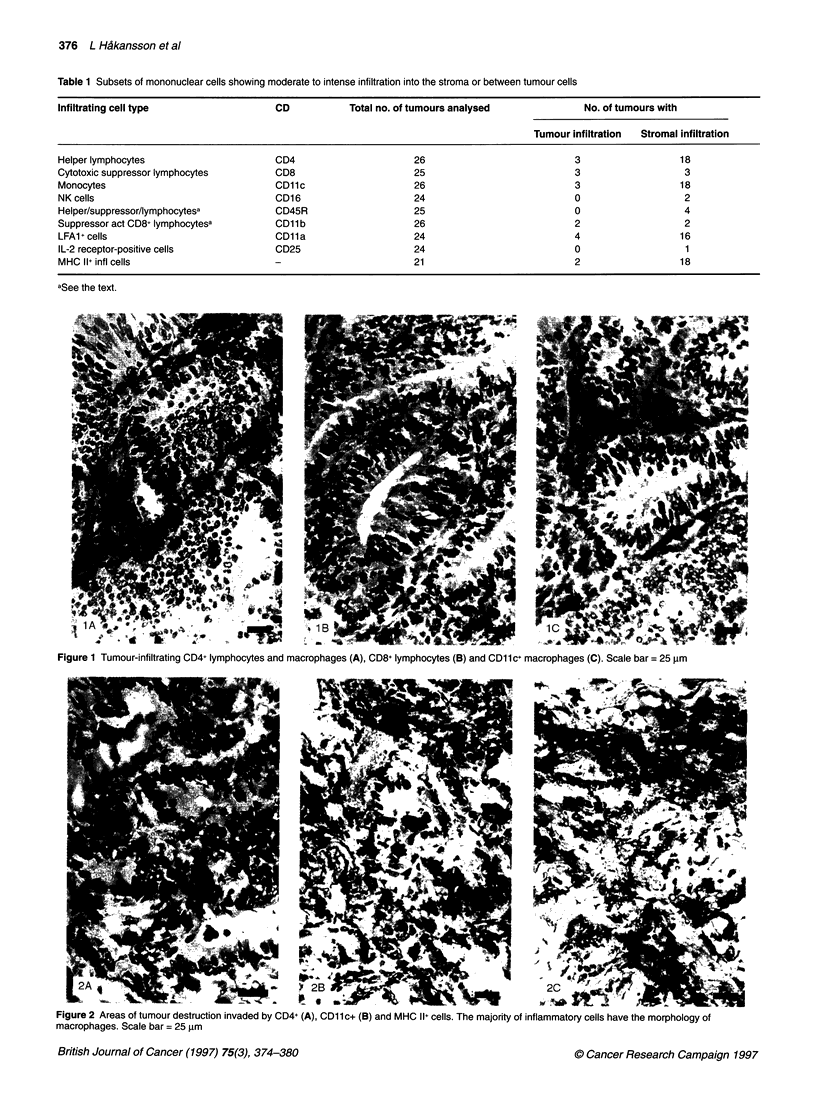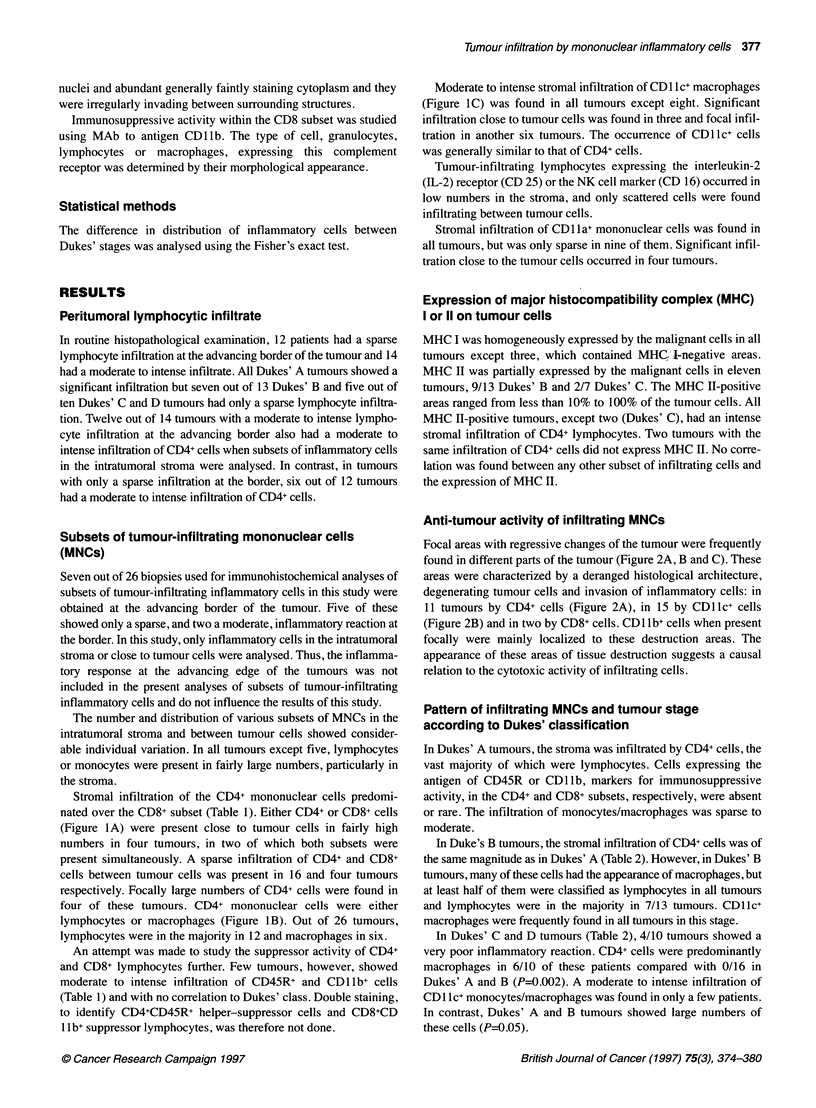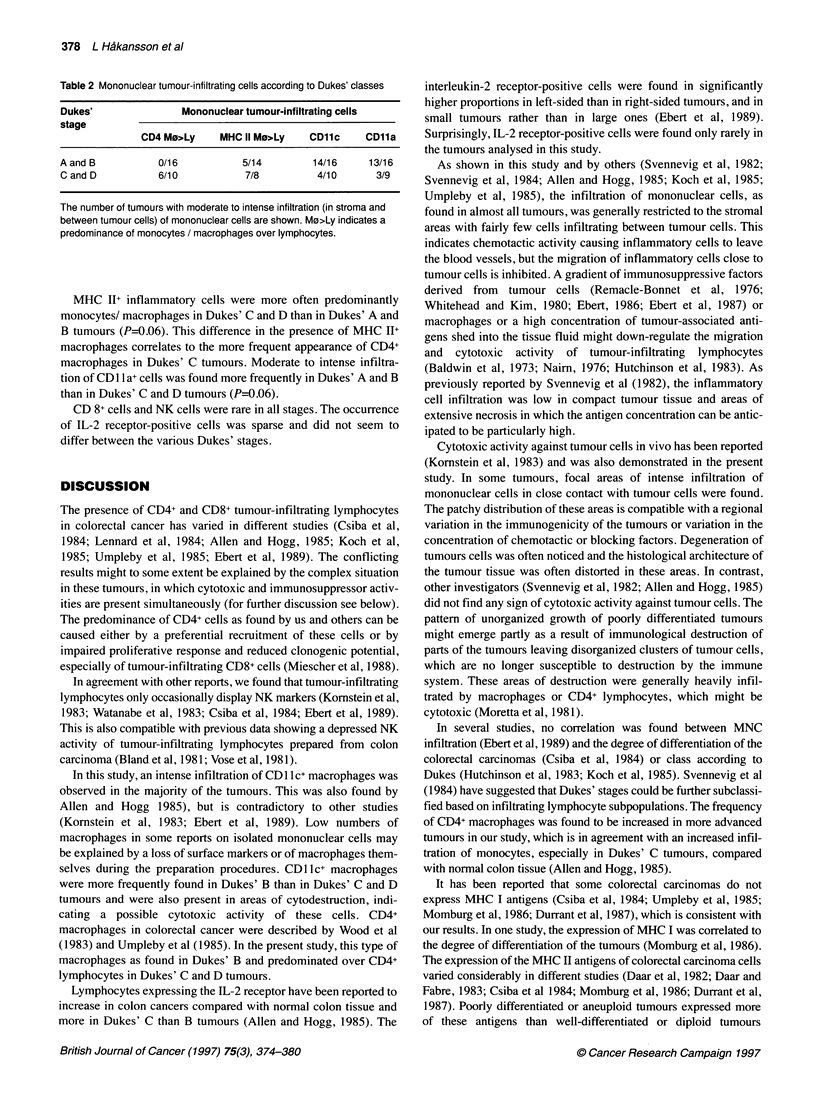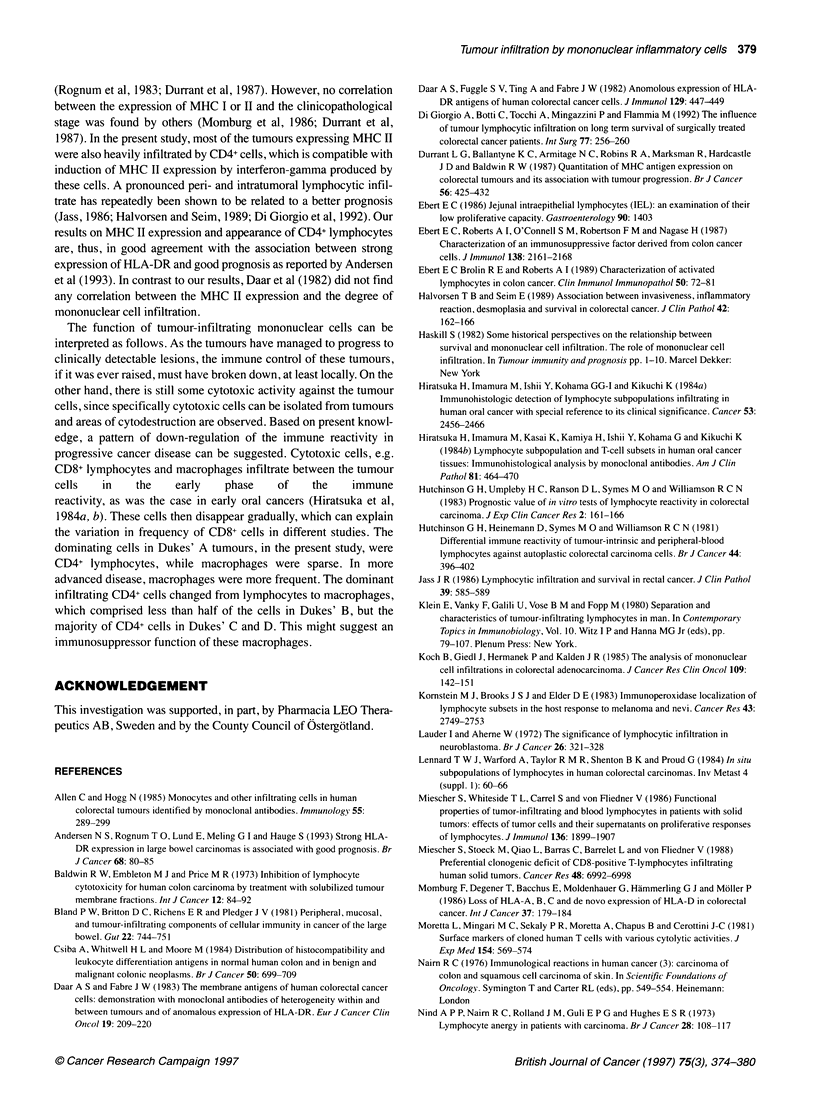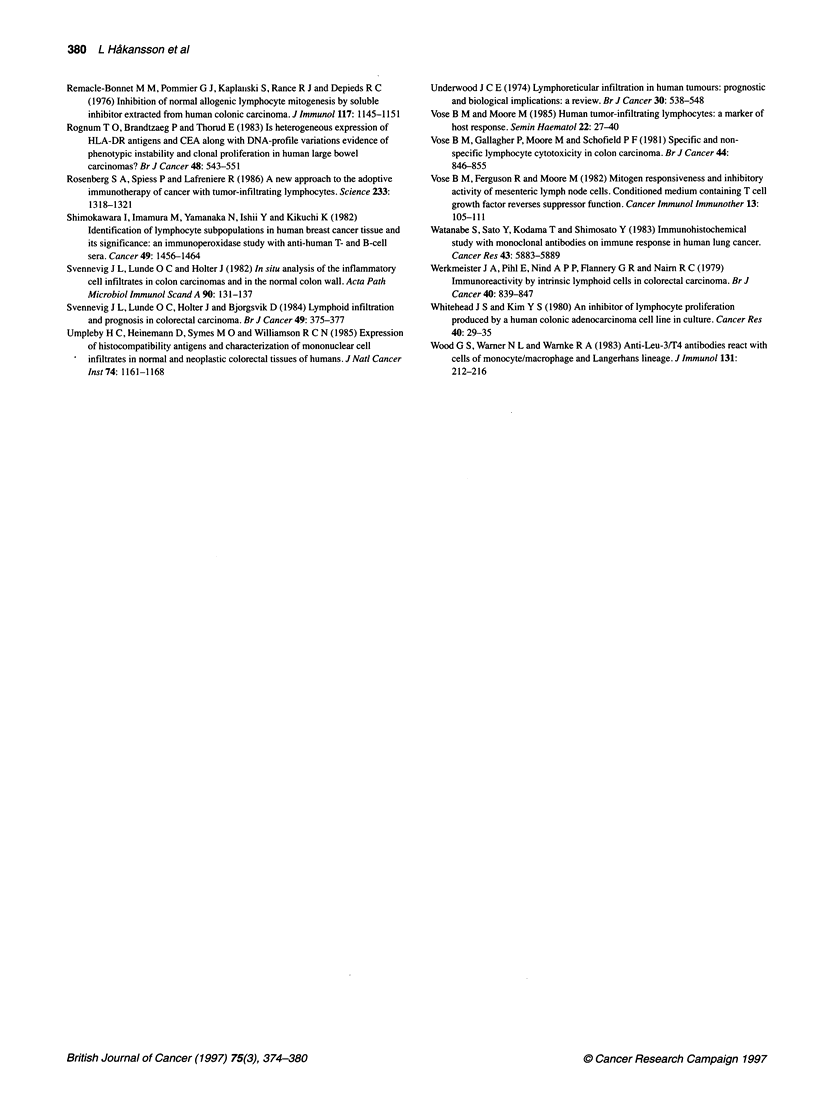# Infiltration of mononuclear inflammatory cells into primary colorectal carcinomas: an immunohistological analysis.

**DOI:** 10.1038/bjc.1997.61

**Published:** 1997

**Authors:** L. Håkansson, G. Adell, B. Boeryd, F. Sjögren, R. Sjödahl

**Affiliations:** Department of Oncology, University Hospital of Linköping, Sweden.

## Abstract

**Images:**


British Journal of Cancer (1997) 75(3), 374–380
© 1997 Cancer Research Campaign

### Infiltration of mononuclear inflammatory cells into primary colorectal carcinomas: an immunohistological analysis

**L Håkansson[1], G Adell[1], B Boeryd[2], F Sjögren[3] and R Sjödahl[4]**

Departments of [1]Oncology, [2]Pathology, [3]Clinical Immunology and [4]Surgery, University Hospital of Linköping, S-581 85 Linköping, Sweden

**Summary** Local immunoregulation mediated by mononuclear tumour-infiltrating cells is considered of importance for tumour progression of colorectal cancer, although the balance between immunosuppressor and cytotoxic activities is unclear. Colorectal cancers from 26 patients were investigated using a panel of monoclonal antibodies in order to identify subsets of mononuclear inflammatory cells and to study their pattern of distribution in relation to tumour stage and cytotoxic immune reactivity against the tumour. In all but five tumours, mononuclear cells, lymphocytes or monocytes were present in fairly large numbers, particularly in the stroma. The infiltration of CD4+ mononuclear cells predominated over the CD8+ subset. Infiltration near the tumour cells was found in four cancers only. Stromal infiltration of CD11c+ macrophages was found in all but eight tumours. Small regressive areas, in which the histological architecture of the tumours was broken down, were found in 17 tumours with intense or moderate infiltration by CD4+ lymphocytes or CD11c+ macrophages. Probably this destruction of tumour tissue was caused by cytotoxic activity of the tumour-infiltrating mononuclear cells. In Dukes' class A and B tumours, CD4+ lymphocytes predominated over CD4+ cells with macrophage morphology, but the latter were increasingly found in Dukes' class C and D disease. The occurrence of MHC II-positive macrophages and lymphocytes in different Dukes' classes was similar to that of CD4+ cells. In contrast to this, CD11c+ and CD11a+ cells were more frequent in Dukes' A and B class tumours compared with Dukes' C and D. Four out of nine tumours of the latter stages showed a poor inflammatory reaction. The interpretation of our results is that the subsets of tumour-infiltrating mononuclear cells change with advancing Dukes' class and that the local immune control is gradually broken down in progressive tumour growth, even if some cytotoxic activity is still present.

**Keywords:** tumour-infiltrating mononuclear cells; colorectal cancer; Dukes' classification

Immune reactivity to colorectal cancer has been suggested by an association between a pronounced peritumoral lymphocytic infiltrate and an improved prognosis (Jass 1986; Halvorsen and Seim, 1989; Di Giorgio et al, 1992). However, the immune defence, if it was ever raised, appears to have deteriorated at least locally by the time tumours develop into clinically detectable lesions. This is in good agreement with the generally found suppression of various immune activities of tumour-infiltrating mononuclear cells (MNCs) (Nind et al, 1973; Klein et al, 1980; Bland et al, 1981; Hutchinson et al, 1981; Vose et al, 1981, 1982; Vose and Moore, 1985; Miescher et al, 1986). Such immunosuppression may be mediated by tumour-derived factors or subsets of MNC.

Some cytotoxic activity against malignant cells might, however, still be present in primary tumours as the degree of mononuclear cell infiltration has been found to correlate with a favourable prognosis in colorectal cancer (Svennevig et al 1984) and some other tumours (Haskil, 1982; Hutchinson et al, 1983; Lauder and Aherne, 1972; Shimokawara et al, 1982; Underwood, 1974; Werkmeister et al, 1979). This may indicate that the local immunosuppression is reversible (Klein et al, 1980; Hutchinson et al, 1981; Vose and Moore, 1985; Rosenberg et al, 1986).

*Received 16 November 1995*
*Revised 22 May 1996*
*Accepted 22 August 1996*

*Correspondence to:* L Håkansson

An increased number of lymphocytes was found in colon cancers compared with normal colons (Svennevig et al, 1982; Allen and Hogg, 1985). These cells were mainly located to the stroma and not close to tumour cells (Svennevig et al, 1982; Allen and Hogg, 1985; Koch et al, 1985; Umpleby et al, 1985). Eighty per cent of the lymphocytes expressed T cell characteristics, 17% B cell characteristics and 6% were null cells (Ebert et al, 1989). The ratio between helper/inducer cells (CD4+) and cytotoxic/suppressor cells (CD8+ in colorectal cancers has varied considerably in different studies (Csiba et al, 1984; Lennard et al, 1984; Allen and Hogg, 1985; Koch et al, 1985; Umpleby et al, 1985; Ebert et al, 1989).

Natural killer cells (NK cells) were either not found or were present in low numbers (Kornstein et al, 1983; Watanabe et al, 1983; Csiba et al, 1984; Koch et al, 1985; Ebert et al, 1989). This is compatible with a depressed NK activity of tumour-infiltrating lymphocytes prepared from colon carcinoma compared with the activity found in autologous blood T cells from these patients (Bland et al, 1981; Vose et al, 1981). The number of macrophages in tumours has varied from sparse (Kornstein et al, 1983; Csiba et al, 1984; Ebert et al, 1989) to frequent (Watanabe et al, 1983; Allen and Hogg, 1985).

Results from immunohistological studies are thus conflicting, and information on immunosuppressor and cytotoxic activities of tumour-infiltrating MNCs in colorectal cancer is almost lacking.

The aim of the present investigation was to use monoclonal antibodies to identify subsets of tumour-infiltrating mononuclear cells and study their pattern of distribution in relation to regressive tumour areas and Dukes' class.

### MATERIAL AND METHODS

### Patients

This report includes 22 primary colon and four rectal carcinomas from ten males and 16 females. Median age was 71 years (range 57–84). The number of tumours according to Dukes' classification was: A 3, B 13, C 5 and D 5. The majority of these tumours were moderately well differentiated. All Dukes' A, 2/13 Dukes' B and no Dukes' C tumours were well differentiated and one poorly differentiated tumour was found in each of Dukes' classes B, C and D.

### Tumour preparation

Surgical specimens were obtained from freshly resected colorectal carcinomas. The tumour tissue was immediately brought to the laboratory, cut into 1 × 1 cm pieces, embedded in OCT compound (Histo-Lab, Göteborg, Sweden), snap frozen in liquid nitrogen and stored at –70°C until further processed. Seven out of 26 biopsies used for immunohistochemical analyses of subsets of tumour-infiltrating inflammatory cells in this study were obtained at the advancing border of the tumour.

### Monoclonal antibodies

*CD4 (Leu-3a, Beckton-Dickinson)*
The antigen is present on helper/inducer T subset lymphocytes and in low density on monocytes and in the cytoplasm of monocytes and macrophages. Dilution of the antibody was 1:25.

*CD8 (Leu-2a, Beckton-Dickinson)*
The antigen is present on cytotoxic/suppressor lymphocytes. The antigen is also expressed on some Leu-11$^+$ cytotoxic NK cells, on a subpopulation of Leu-7$^+$ cells (which do not have cytotoxic and NK activity), on some Leu-8$^+$ cells (which participate in suppression of B cell function) and on Leu-15$^+$ cells, which are associated with suppressor function. Dilution of the antibody was 1:50.

*CD45R (2H4, Coulter, Stockholm, Sweden)*
The antigen is expressed on CD4$^+$ and CD8$^+$ T lymphocytes. It is present on some B and null cells. CD4-positive cells, which express this antigen, belong to the suppressor/inducer subset. Dilution of the antibody was 1:100.

*CD11b (CR-3, Leu-15 Beckton-Dickinson)*
The antigen is present on approximately 30% of peripheral blood lymphocytes and 90% of NK cells, neutrophils, eosinophils and monocytes. CD8-positive cells expressing this antigen are associated with suppression. Dilution of the antibody was 1:25.

*CD11c (M-5, Beckton-Dickinson)*
The antigen is present on monocytes and in low density on granulocytes and large granular lymphocytes in peripheral blood. It is also expressed on macrophages in normal lymphoid tissue and on Kupffer cells in liver and alveolar macrophages in lung tissue. Dilution of the antibody was 1:5.

*CD25 (IL-2 receptor, Beckton-Dickinson)*
The antigen, Tac-antigen or human receptor for interleukin-2 is present on activated T cells, B cells, NK cells and some macrophages. Dilution of the antibody was 1:25.

*CD16 (Leu-7, Beckton-Dickinson)*
The antigen is present on human NK cells and neutrophils. It is associated with the Fc receptor for IgG. The antibody was undiluted.

*CD11a (Immunotech, Stockholm, Sweden)*
The antigen, alpha-chain of the LFA-1 molecule, is present on granulocytes and macrophages. Dilution of the antibody was 1:100.

*MHC I (HLA-ABC, Dakopatts)*
The antibody is directed against a monomorphic epitope on the 45-kDa polypeptide products of the HLA-A, -B and -C loci. Dilution of the antibody was 1:100.

*MHC II (HLA-DR, Beckton-Dickinson)*
The antigen is expressed on B lymphocytes, monocytes/macrophages, activated T cells and some tumour cells. It is co-expressed with anti-Leu-6 on Langerhans cells of the epidermis. Dilution of the antibody was 1:50.

### Immunological staining

The biopsies were cut into 6-μm sections and placed on multispot slides (Novakemi, Stockholm, Sweden) coated with a 0.5% gelatine solution. The sections were airdried for 3–18 h before staining and fixed for 10 min in acetone at 20°C. After drying, the sections were incubated with monoclonal antibodies (see above) for 30 min. Mouse IgG (Sigma, Stockholm, Sweden) was used as a negative control. After washing in phosphate-buffered saline (PBS) twice for 5 min, the sections were incubated with rabbit anti-mouse immunoglobulins (Dakopatts), washed and incubated with PAP mouse (Dakopatts). All dilutions were made in 0.5% PBS–HSA. All incubation times were 30 min. After washing, the slides were treated with 0.05% DAB (Sigma, Stockholm, Sweden) in 0.001% hydrogen peroxide. Endogenous peroxidase activity was not blocked before the staining procedure. Human tonsils were used as a positive control. The slides were counterstained in Mayer's haematoxylin and mounted in Aquamount (Gurr, Malmö, Sweden).

### Evaluation of peritumoral lymphocytic infiltrate

Routine, haemtoxylin and eosin-stained histopathological specimens were used to determine the infiltration of lymphocytes at the advancing border of the tumours. The degree of the infiltrate was scored as sparse or moderate/intense.

### Evaluation of mononuclear cells

The distribution of infiltrating cells was often heterogeneous, and counting of cells per microscopic field was not performed. Instead, the overall occurrence of each subset of these cells in the intratumoral stroma and intermingled between tumour cells was scored as: – (absent), + (sparse, low numbers), ++ (moderate) and +++ (intense), independently by two investigators having no information about the patients or the result of routine histological examination. Inflammatory cells of necrotic areas were not registered in this study. There was a more than 90% agreement in the analyses between the two investigators.

CD4$^+$ mononuclear cells, lymphocytes or macrophages were identified by their morphological appearance. Cells scored as lymphocytes had small nuclei and sparse cytoplasm with distinct cell membrane. In contrast, the macrophages displayed large

© Cancer Research Campaign 1997

**Table 1** Subsets of mononuclear cells showing moderate to intense infiltration into the stroma or between tumour cells

| Infiltrating cell type | CD | Total no. of tumours analysed | No. of tumours with | |
|---|---|---|---|---|
| | | | Tumour infiltration | Stromal infiltration |
| Helper lymphocytes | CD4 | 26 | 3 | 18 |
| Cytotoxic suppressor lymphocytes | CD8 | 25 | 3 | 3 |
| Monocytes | CD11c | 26 | 3 | 18 |
| NK cells | CD16 | 24 | 0 | 2 |
| Helper/suppressor/lymphocytes[a] | CD45R | 25 | 0 | 4 |
| Suppressor act CD8$^+$ lymphocytes[a] | CD11b | 26 | 2 | 2 |
| LFA1$^+$ cells | CD11a | 24 | 4 | 16 |
| IL-2 receptor-positive cells | CD25 | 24 | 0 | 1 |
| MHC II$^+$ infl cells | – | 21 | 2 | 18 |

[a]See the text.

**Figure 1** Tumour-infiltrating CD4$^+$ lymphocytes and macrophages (**A**), CD8$^+$ lymphocytes (**B**) and CD11c$^+$ macrophages (**C**). Scale bar = 25 μm

**Figure 2** Areas of tumour destruction invaded by CD4$^+$ (**A**), CD11c+ (**B**) and MHC II$^+$ cells. The majority of inflammatory cells have the morphology of macrophages. Scale bar = 25 μm

*© Cancer Research Campaign 1997*

nuclei and abundant generally faintly staining cytoplasm and they were irregularly invading between surrounding structures.

Immunosuppressive activity within the CD8 subset was studied using MAb to antigen CD11b. The type of cell, granulocytes, lymphocytes or macrophages, expressing this complement receptor was determined by their morphological appearance.

### Statistical methods

The difference in distribution of inflammatory cells between Dukes' stages was analysed using the Fisher's exact test.

### RESULTS

### Peritumoral lymphocytic infiltrate

In routine histopathological examination, 12 patients had a sparse lymphocyte infiltration at the advancing border of the tumour and 14 had a moderate to intense infiltrate. All Dukes' A tumours showed a significant infiltration but seven out of 13 Dukes' B and five out of ten Dukes' C and D tumours had only a sparse lymphocyte infiltration. Twelve out of 14 tumours with a moderate to intense lymphocyte infiltration at the advancing border also had a moderate to intense infiltration of $CD4^+$ cells when subsets of inflammatory cells in the intratumoral stroma were analysed. In contrast, in tumours with only a sparse infiltration at the border, six out of 12 tumours had a moderate to intense infiltration of $CD4^+$ cells.

### Subsets of tumour-infiltrating mononuclear cells (MNCs)

Seven out of 26 biopsies used for immunohistochemical analyses of subsets of tumour-infiltrating inflammatory cells in this study were obtained at the advancing border of the tumour. Five of these showed only a sparse, and two a moderate, inflammatory reaction at the border. In this study, only inflammatory cells in the intratumoral stroma or close to tumour cells were analysed. Thus, the inflammatory response at the advancing edge of the tumours was not included in the present analyses of subsets of tumour-infiltrating inflammatory cells and do not influence the results of this study.

The number and distribution of various subsets of MNCs in the intratumoral stroma and between tumour cells showed considerable individual variation. In all tumours except five, lymphocytes or monocytes were present in fairly large numbers, particularly in the stroma.

Stromal infiltration of the $CD4^+$ mononuclear cells predominated over the $CD8^+$ subset (Table 1). Either $CD4^+$ or $CD8^+$ cells (Figure 1A) were present close to tumour cells in fairly high numbers in four tumours, in two of which both subsets were present simultaneously. A sparse infiltration of $CD4^+$ and $CD8^+$ cells between tumour cells was present in 16 and four tumours respectively. Focally large numbers of $CD4^+$ cells were found in four of these tumours. $CD4^+$ mononuclear cells were either lymphocytes or macrophages (Figure 1B). Out of 26 tumours, lymphocytes were in the majority in 12 and macrophages in six.

An attempt was made to study the suppressor activity of $CD4^+$ and $CD8^+$ lymphocytes further. Few tumours, however, showed moderate to intense infiltration of $CD45R^+$ and $CD11b^+$ cells (Table 1) and with no correlation to Dukes' class. Double staining, to identify $CD4^+CD45R^+$ helper–suppressor cells and $CD8^+CD$ $11b^+$ suppressor lymphocytes, was therefore not done.

Moderate to intense stromal infiltration of $CD11c^+$ macrophages (Figure 1C) was found in all tumours except eight. Significant infiltration close to tumour cells was found in three and focal infiltration in another six tumours. The occurrence of $CD11c^+$ cells was generally similar to that of $CD4^+$ cells.

Tumour-infiltrating lymphocytes expressing the interleukin-2 (IL-2) receptor (CD 25) or the NK cell marker (CD 16) occurred in low numbers in the stroma, and only scattered cells were found infiltrating between tumour cells.

Stromal infiltration of $CD11a^+$ mononuclear cells was found in all tumours, but was only sparse in nine of them. Significant infiltration close to the tumour cells occurred in four tumours.

### Expression of major histocompatibility complex (MHC) I or II on tumour cells

MHC I was homogeneously expressed by the malignant cells in all tumours except three, which contained MHC I-negative areas. MHC II was partially expressed by the malignant cells in eleven tumours, 9/13 Dukes' B and 2/7 Dukes' C. The MHC II-positive areas ranged from less than 10% to 100% of the tumour cells. All MHC II-positive tumours, except two (Dukes' C), had an intense stromal infiltration of $CD4^+$ lymphocytes. Two tumours with the same infiltration of $CD4^+$ cells did not express MHC II. No correlation was found between any other subset of infiltrating cells and the expression of MHC II.

### Anti-tumour activity of infiltrating MNCs

Focal areas with regressive changes of the tumour were frequently found in different parts of the tumour (Figure 2A, B and C). These areas were characterized by a deranged histological architecture, degenerating tumour cells and invasion of inflammatory cells: in 11 tumours by $CD4^+$ cells (Figure 2A), in 15 by $CD11c^+$ cells (Figure 2B) and in two by $CD8^+$ cells. $CD11b^+$ cells when present focally were mainly localized to these destruction areas. The appearance of these areas of tissue destruction suggests a causal relation to the cytotoxic activity of infiltrating cells.

### Pattern of infiltrating MNCs and tumour stage according to Dukes' classification

In Dukes' A tumours, the stroma was infiltrated by $CD4^+$ cells, the vast majority of which were lymphocytes. Cells expressing the antigen of CD45R or CD11b, markers for immunosuppressive activity, in the $CD4^+$ and $CD8^+$ subsets, respectively, were absent or rare. The infiltration of monocytes/macrophages was sparse to moderate.

In Duke's B tumours, the stromal infiltration of $CD4^+$ cells was of the same magnitude as in Dukes' A (Table 2). However, in Dukes' B tumours, many of these cells had the appearance of macrophages, but at least half of them were classified as lymphocytes in all tumours and lymphocytes were in the majority in 7/13 tumours. $CD11c^+$ macrophages were frequently found in all tumours in this stage.

In Dukes' C and D tumours (Table 2), 4/10 tumours showed a very poor inflammatory reaction. $CD4^+$ cells were predominantly macrophages in 6/10 of these patients compared with 0/16 in Dukes' A and B ($P$=0.002). A moderate to intense infiltration of $CD11c^+$ monocytes/macrophages was found in only a few patients. In contrast, Dukes' A and B tumours showed large numbers of these cells ($P$=0.05).

© Cancer Research Campaign 1997

**Table 2** Mononuclear tumour-infiltrating cells according to Dukes' classes

| Dukes' stage | Mononuclear tumour-infiltrating cells | | | |
|---|---|---|---|---|
| | CD4 Mø>Ly | MHC II Mø>Ly | CD11c | CD11a |
| A and B | 0/16 | 5/14 | 14/16 | 13/16 |
| C and D | 6/10 | 7/8 | 4/10 | 3/9 |

The number of tumours with moderate to intense infiltration (in stroma and between tumour cells) of mononuclear cells are shown. Mø>Ly indicates a predominance of monocytes / macrophages over lymphocytes.

MHC II$^+$ inflammatory cells were more often predominantly monocytes/ macrophages in Dukes' C and D than in Dukes' A and B tumours ($P$=0.06). This difference in the presence of MHC II$^+$ macrophages correlates to the more frequent appearance of CD4$^+$ macrophages in Dukes' C tumours. Moderate to intense infiltration of CD11a$^+$ cells was found more frequently in Dukes' A and B than in Dukes' C and D tumours ($P$=0.06).

CD 8$^+$ cells and NK cells were rare in all stages. The occurrence of IL-2 receptor-positive cells was sparse and did not seem to differ between the various Dukes' stages.

### DISCUSSION

The presence of CD4$^+$ and CD8$^+$ tumour-infiltrating lymphocytes in colorectal cancer has varied in different studies (Csiba et al, 1984; Lennard et al, 1984; Allen and Hogg, 1985; Koch et al, 1985; Umpleby et al, 1985; Ebert et al, 1989). The conflicting results might to some extent be explained by the complex situation in these tumours, in which cytotoxic and immunosuppressor activities are present simultaneously (for further discussion see below). The predominance of CD4$^+$ cells as found by us and others can be caused either by a preferential recruitment of these cells or by impaired proliferative response and reduced clonogenic potential, especially of tumour-infiltrating CD8$^+$ cells (Miescher et al, 1988).

In agreement with other reports, we found that tumour-infiltrating lymphocytes only occasionally display NK markers (Kornstein et al, 1983; Watanabe et al, 1983; Csiba et al, 1984; Ebert et al, 1989). This is also compatible with previous data showing a depressed NK activity of tumour-infiltrating lymphocytes prepared from colon carcinoma (Bland et al, 1981; Vose et al, 1981).

In this study, an intense infiltration of CD11c$^+$ macrophages was observed in the majority of the tumours. This was also found by Allen and Hogg 1985), but is contradictory to other studies (Kornstein et al, 1983; Ebert et al, 1989). Low numbers of macrophages in some reports on isolated mononuclear cells may be explained by a loss of surface markers or of macrophages themselves during the preparation procedures. CD11c$^+$ macrophages were more frequently found in Dukes' B than in Dukes' C and D tumours and were also present in areas of cytodestruction, indicating a possible cytotoxic activity of these cells. CD4$^+$ macrophages in colorectal cancer were described by Wood et al (1983) and Umpleby et al (1985). In the present study, this type of macrophages as found in Dukes' B and predominated over CD4$^+$ lymphocytes in Dukes' C and D tumours.

Lymphocytes expressing the IL-2 receptor have been reported to increase in colon cancers compared with normal colon tissue and more in Dukes' C than B tumours (Allen and Hogg, 1985). The interleukin-2 receptor-positive cells were found in significantly higher proportions in left-sided than in right-sided tumours, and in small tumours rather than in large ones (Ebert et al, 1989). Surprisingly, IL-2 receptor-positive cells were found only rarely in the tumours analysed in this study.

As shown in this study and by others (Svennevig et al, 1982; Svennevig et al, 1984; Allen and Hogg, 1985; Koch et al, 1985; Umpleby et al, 1985), the infiltration of mononuclear cells, as found in almost all tumours, was generally restricted to the stromal areas with fairly few cells infiltrating between tumour cells. This indicates chemotactic activity causing inflammatory cells to leave the blood vessels, but the migration of inflammatory cells close to tumour cells is inhibited. A gradient of immunosuppressive factors derived from tumour cells (Remacle-Bonnet et al, 1976; Whitehead and Kim, 1980; Ebert, 1986; Ebert et al, 1987) or macrophages or a high concentration of tumour-associated antigens shed into the tissue fluid might down-regulate the migration and cytotoxic activity of tumour-infiltrating lymphocytes (Baldwin et al, 1973; Nairn, 1976; Hutchinson et al, 1983). As previously reported by Svennevig et al (1982), the inflammatory cell infiltration was low in compact tumour tissue and areas of extensive necrosis in which the antigen concentration can be anticipated to be particularly high.

Cytotoxic activity against tumour cells in vivo has been reported (Kornstein et al, 1983) and was also demonstrated in the present study. In some tumours, focal areas of intense infiltration of mononuclear cells in close contact with tumour cells were found. The patchy distribution of these areas is compatible with a regional variation in the immunogenicity of the tumours or variation in the concentration of chemotactic or blocking factors. Degeneration of tumours cells was often noticed and the histological architecture of the tumour tissue was often distorted in these areas. In contrast, other investigators (Svennevig et al, 1982; Allen and Hogg, 1985) did not find any sign of cytotoxic activity against tumour cells. The pattern of unorganized growth of poorly differentiated tumours might emerge partly as a result of immunological destruction of parts of the tumours leaving disorganized clusters of tumour cells, which are no longer susceptible to destruction by the immune system. These areas of destruction were generally heavily infiltrated by macrophages or CD4$^+$ lymphocytes, which might be cytotoxic (Moretta et al, 1981).

In several studies, no correlation was found between MNC infiltration (Ebert et al, 1989) and the degree of differentiation of the colorectal carcinomas (Csiba et al, 1984) or class according to Dukes (Hutchinson et al, 1983; Koch et al, 1985). Svennevig et al (1984) have suggested that Dukes' stages could be further subclassified based on infiltrating lymphocyte subpopulations. The frequency of CD4$^+$ macrophages was found to be increased in more advanced tumours in our study, which is in agreement with an increased infiltration of monocytes, especially in Dukes' C tumours, compared with normal colon tissue (Allen and Hogg, 1985).

It has been reported that some colorectal carcinomas do not express MHC I antigens (Csiba et al, 1984; Umpleby et al, 1985; Momburg et al, 1986; Durrant et al, 1987), which is consistent with our results. In one study, the expression of MHC I was correlated to the degree of differentiation of the tumours (Momburg et al, 1986). The expression of the MHC II antigens of colorectal carcinoma cells varied considerably in different studies (Daar et al, 1982; Daar and Fabre, 1983; Csiba et al 1984; Momburg et al, 1986; Durrant et al, 1987). Poorly differentiated or aneuploid tumours expressed more of these antigens than well-differentiated or diploid tumours

 © Cancer Research Campaign 1997

(Rognum et al, 1983; Durrant et al, 1987). However, no correlation between the expression of MHC I or II and the clinicopathological stage was found by others (Momburg et al, 1986; Durrant et al, 1987). In the present study, most of the tumours expressing MHC II were also heavily infiltrated by CD4+ cells, which is compatible with induction of MHC II expression by interferon-gamma produced by these cells. A pronounced peri- and intratumoral lymphocytic infiltrate has repeatedly been shown to be related to a better prognosis (Jass, 1986; Halvorsen and Seim, 1989; Di Giorgio et al, 1992). Our results on MHC II expression and appearance of CD4+ lymphocytes are, thus, in good agreement with the association between strong expression of HLA-DR and good prognosis as reported by Andersen et al (1993). In contrast to our results, Daar et al (1982) did not find any correlation between the MHC II expression and the degree of mononuclear cell infiltration.

The function of tumour-infiltrating mononuclear cells can be interpreted as follows. As the tumours have managed to progress to clinically detectable lesions, the immune control of these tumours, if it was ever raised, must have broken down, at least locally. On the other hand, there is still some cytotoxic activity against the tumour cells, since specifically cytotoxic cells can be isolated from tumours and areas of cytodestruction are observed. Based on present knowledge, a pattern of down-regulation of the immune reactivity in progressive cancer disease can be suggested. Cytotoxic cells, e.g. CD8+ lymphocytes and macrophages infiltrate between the tumour cells in the early phase of the immune reactivity, as was the case in early oral cancers (Hiratsuka et al, 1984*a, b*). These cells then disappear gradually, which can explain the variation in frequency of CD8+ cells in different studies. The dominating cells in Dukes' A tumours, in the present study, were CD4+ lymphocytes, while macrophages were sparse. In more advanced disease, macrophages were more frequent. The dominant infiltrating CD4+ cells changed from lymphocytes to macrophages, which comprised less than half of the cells in Dukes' B, but the majority of CD4+ cells in Dukes' C and D. This might suggest an immunosuppressor function of these macrophages.

### ACKNOWLEDGEMENT

This investigation was supported, in part, by Pharmacia LEO Therapeutics AB, Sweden and by the County Council of Östergötland.

### REFERENCES

Allen C and Hogg N (1985) Monocytes and other infiltrating cells in human colorectal tumours identified by monoclonal antibodies. *Immunology* **55**: 289–299

Andersen N S, Rognum T O, Lund E, Meling G I and Hauge S (1993) Strong HLA-DR expression in large bowel carcinomas is associated with good prognosis. *Br J Cancer* **68**: 80–85

Baldwin R W, Embleton M J and Price M R (1973) Inhibition of lymphocyte cytotoxicity for human colon carcinoma by treatment with solubilized tumour membrane fractions. *Int J Cancer* **12**: 84–92

Bland P W, Britton D C, Richens E R and Pledger J V (1981) Peripheral, mucosal, and tumour-infiltrating components of cellular immunity in cancer of the large bowel. *Gut* **22**: 744–751

Csiba A, Whitwell H L and Moore M (1984) Distribution of histocompatibility and leukocyte differentiation antigens in normal human colon and in benign and malignant colonic neoplasms. *Br J Cancer* **50**: 699–709

Daar A S and Fabre J W (1983) The membrane antigens of human colorectal cancer cells: demonstration with monoclonal antibodies of heterogeneity within and between tumours and of anomalous expression of HLA-DR. *Eur J Cancer Clin Oncol* **19**: 209–220

Daar A S, Fuggle S V, Ting A and Fabre J W (1982) Anomolous expression of HLA-DR antigens of human colorectal cancer cells. *J Immunol* **129**: 447–449

Di Giorgio A, Botti C, Tocchi A, Mingazzini P and Flammia M (1992) The influence of tumour lymphocytic infiltration on long term survival of surgically treated colorectal cancer patients. *Int Surg* **77**: 256–260

Durrant L G, Ballantyne K C, Armitage N C, Robins R A, Marksman R, Hardcastle J D and Baldwin R W (1987) Quantitation of MHC antigen expression on colorectal tumours and its association with tumour progression. *Br J Cancer* **56**: 425–432

Ebert E C (1986) Jejunal intraepithelial lymphocytes (IEL): an examination of their low proliferative capacity. *Gastroenterology* **90**: 1403

Ebert E C, Roberts A I, O'Connell S M, Robertson F M and Nagase H (1987) Characterization of an immunosuppressive factor derived from colon cancer cells. *J Immunol* **138**: 2161–2168

Ebert E C Brolin R E and Roberts A I (1989) Characterization of activated lymphocytes in colon cancer. *Clin Immunol Immunopathol* **50**: 72–81

Halvorsen T B and Seim E (1989) Association between invasiveness, inflammatory reaction, desmoplasia and survival in colorectal cancer. *J Clin Pathol* **42**: 162–166

Haskill S (1982) Some historical perspectives on the relationship between survival and mononuclear cell infiltration. The role of mononuclear cell infiltration. In *Tumour immunity and prognosis* pp. 1–10. Marcel Dekker: New York

Hiratsuka H, Imamura M, Ishii Y, Kohama GG-I and Kikuchi K (1984*a*) Immunohistologic detection of lymphocyte subpopulations infiltrating in human oral cancer with special reference to its clinical significance. *Cancer* **53**: 2456–2466

Hiratsuka H, Imamura M, Kasai K, Kamiya H, Ishii Y, Kohama G and Kikuchi K (1984*b*) Lymphocyte subpopulation and T-cell subsets in human oral cancer tissues: Immunohistological analysis by monoclonal antibodies. *Am J Clin Pathol* **81**: 464–470

Hutchinson G H, Umpleby H C, Ranson D L, Symes M O and Williamson R C N (1983) Prognostic value of *in vitro* tests of lymphocyte reactivity in colorectal carcinoma. *J Exp Clin Cancer Res* **2**: 161–166

Hutchinson G H, Heinemann D, Symes M O and Williamson R C N (1981) Differential immune reactivity of tumour-intrinsic and peripheral-blood lymphocytes against autoplastic colorectal carcinoma cells. *Br J Cancer* **44**: 396–402

Jass J R (1986) Lymphocytic infiltration and survival in rectal cancer. *J Clin Pathol* **39**: 585–589

Klein E, Vanky F, Galili U, Vose B M and Fopp M (1980) Separation and characteristics of tumour-infiltrating lymphocytes in man. In *Contemporary Topics in Immunobiology*, Vol. 10. Witz I P and Hanna MG Jr (eds), pp. 79–107. Plenum Press: New York.

Koch B, Giedl J, Hermanek P and Kalden J R (1985) The analysis of mononuclear cell infiltrations in colorectal adenocarcinoma. *J Cancer Res Clin Oncol* **109**: 142–151

Kornstein M J, Brooks J S J and Elder D E (1983) Immunoperoxidase localization of lymphocyte subsets in the host response to melanoma and nevi. *Cancer Res* **43**: 2749–2753

Lauder I and Aherne W (1972) The significance of lymphocytic infiltration in neuroblastoma. *Br J Cancer* **26**: 321–328

Lennard T W J, Warford A, Taylor R M R, Shenton B K and Proud G (1984) *In situ* subpopulations of lymphocytes in human colorectal carcinomas. Inv Metast 4 (suppl. 1): 60–66

Miescher S, Whiteside T L, Carrel S and von Fliedner V (1986) Functional properties of tumor-infiltrating and blood lymphocytes in patients with solid tumors: effects of tumor cells and their supernatants on proliferative responses of lymphocytes. *J Immunol* **136**: 1899–1907

Miescher S, Stoeck M, Qiao L, Barras C, Barrelet L and von Fliedner V (1988) Preferential clonogenic deficit of CD8-positive T-lymphocytes infiltrating human solid tumors. *Cancer Res* **48**: 6992–6998

Momburg F, Degener T, Bacchus E, Moldenhauer G, Hämmerling G J and Möller P (1986) Loss of HLA-A, B, C and de novo expression of HLA-D in colorectal cancer. *Int J Cancer* **37**: 179–184

Moretta L, Mingari M C, Sekaly P R, Moretta A, Chapus B and Cerottini J-C (1981) Surface markers of cloned human T cells with various cytolytic activities. *J Exp Med* **154**: 569–574

Nairn R C (1976) Immunological reactions in human cancer (3): carcinoma of colon and squamous cell carcinoma of skin. In *Scientific Foundations of Oncology*. Symington T and Carter RL (eds), pp. 549–554. Heinemann: London

Nind A P P, Nairn R C, Rolland J M, Guli E P G and Hughes E S R (1973) Lymphocyte anergy in patients with carcinoma. *Br J Cancer* **28**: 108–117

© Cancer Research Campaign 1997 

Remacle-Bonnet M M, Pommier G J, Kaplanski S, Rance R J and Depieds R C (1976) Inhibition of normal allogenic lymphocyte mitogenesis by soluble inhibitor extracted from human colonic carcinoma. *J Immunol* **117**: 1145–1151

Rognum T O, Brandtzaeg P and Thorud E (1983) Is heterogeneous expression of HLA-DR antigens and CEA along with DNA-profile variations evidence of phenotypic instability and clonal proliferation in human large bowel carcinomas? *Br J Cancer* **48**: 543–551

Rosenberg S A, Spiess P and Lafreniere R (1986) A new approach to the adoptive immunotherapy of cancer with tumor-infiltrating lymphocytes. *Science* **233**: 1318–1321

Shimokawara I, Imamura M, Yamanaka N, Ishii Y and Kikuchi K (1982) Identification of lymphocyte subpopulations in human breast cancer tissue and its significance: an immunoperoxidase study with anti-human T- and B-cell sera. *Cancer* **49**: 1456–1464

Svennevig J L, Lunde O C and Holter J (1982) *In situ* analysis of the inflammatory cell infiltrates in colon carcinomas and in the normal colon wall. *Acta Path Microbiol Immunol Scand A* **90**: 131–137

Svennevig J L, Lunde O C, Holter J and Bjorgsvik D (1984) Lymphoid infiltration and prognosis in colorectal carcinoma. *Br J Cancer* **49**: 375–377

Umpleby H C, Heinemann D, Symes M O and Williamson R C N (1985) Expression of histocompatibility antigens and characterization of mononuclear cell infiltrates in normal and neoplastic colorectal tissues of humans. *J Natl Cancer Inst* **74**: 1161–1168

Underwood J C E (1974) Lymphoreticular infiltration in human tumours: prognostic and biological implications: a review. *Br J Cancer* **30**: 538–548

Vose B M and Moore M (1985) Human tumor-infiltrating lymphocytes: a marker of host response. *Semin Haematol* **22**: 27–40

Vose B M, Gallagher P, Moore M and Schofield P F (1981) Specific and non-specific lymphocyte cytotoxicity in colon carcinoma. *Br J Cancer* **44**: 846–855

Vose B M, Ferguson R and Moore M (1982) Mitogen responsiveness and inhibitory activity of mesenteric lymph node cells. Conditioned medium containing T cell growth factor reverses suppressor function. *Cancer Immunol Immunother* **13**: 105–111

Watanabe S, Sato Y, Kodama T and Shimosato Y (1983) Immunohistochemical study with monoclonal antibodies on immune response in human lung cancer. *Cancer Res* **43**: 5883–5889

Werkmeister J A, Pihl E, Nind A P P, Flannery G R and Nairn R C (1979) Immunoreactivity by intrinsic lymphoid cells in colorectal carcinoma. *Br J Cancer* **40**: 839–847

Whitehead J S and Kim Y S (1980) An inhibitor of lymphocyte proliferation produced by a human colonic adenocarcinoma cell line in culture. *Cancer Res* **40**: 29–35

Wood G S, Warner N L and Warnke R A (1983) Anti-Leu-3/T4 antibodies react with cells of monocyte/macrophage and Langerhans lineage. *J Immunol* **131**: 212–216

© Cancer Research Campaign 1997